# Syrian hamsters as a small animal model for SARS-CoV-2 infection and countermeasure development

**DOI:** 10.1073/pnas.2009799117

**Published:** 2020-06-22

**Authors:** Masaki Imai, Kiyoko Iwatsuki-Horimoto, Masato Hatta, Samantha Loeber, Peter J. Halfmann, Noriko Nakajima, Tokiko Watanabe, Michiko Ujie, Kenta Takahashi, Mutsumi Ito, Shinya Yamada, Shufang Fan, Shiho Chiba, Makoto Kuroda, Lizheng Guan, Kosuke Takada, Tammy Armbrust, Aaron Balogh, Yuri Furusawa, Moe Okuda, Hiroshi Ueki, Atsuhiro Yasuhara, Yuko Sakai-Tagawa, Tiago J. S. Lopes, Maki Kiso, Seiya Yamayoshi, Noriko Kinoshita, Norio Ohmagari, Shin-ichiro Hattori, Makoto Takeda, Hiroaki Mitsuya, Florian Krammer, Tadaki Suzuki, Yoshihiro Kawaoka

**Affiliations:** ^a^Division of Virology, Department of Microbiology and Immunology, Institute of Medical Science, University of Tokyo, 108-8639 Tokyo, Japan;; ^b^Influenza Research Institute, Department of Pathobiological Sciences, School of Veterinary Medicine, University of Wisconsin-Madison, Madison, WI 53711;; ^c^Department of Surgical Sciences, School of Veterinary Medicine, University of Wisconsin-Madison, Madison, WI 53706;; ^d^Department of Pathology, National Institute of Infectious Diseases, 162-8640 Tokyo, Japan;; ^e^Disease Control and Prevention Center, National Center for Global Health and Medicine, 162-8655 Tokyo, Japan;; ^f^Department of Virology 3, National Institute of Infectious Diseases, 208-0011 Tokyo, Japan;; ^g^Department of Microbiology, Icahn School of Medicine at Mount Sinai, New York, NY 10029;; ^h^Department of Special Pathogens, International Research Center for Infectious Diseases, Institute of Medical Science, University of Tokyo, 108-8639 Tokyo, Japan

**Keywords:** Syrian hamsters, SARS-CoV-2, infection, countermeasure

## Abstract

At the end of 2019, a novel coronavirus (severe acute respiratory syndrome coronavirus 2; SARS-CoV-2) was detected in Wuhan, China, that spread rapidly around the world, with severe consequences for human health and the global economy. Here, we assessed the replicative ability and pathogenesis of SARS-CoV-2 isolates in Syrian hamsters. SARS-CoV-2 isolates replicated efficiently in the lungs of hamsters, causing severe pathological lung lesions following intranasal infection. In addition, microcomputed tomographic imaging revealed severe lung injury that shared characteristics with SARS-CoV-2−infected human lung, including severe, bilateral, peripherally distributed, multilobular ground glass opacity, and regions of lung consolidation. SARS-CoV-2−infected hamsters mounted neutralizing antibody responses and were protected against subsequent rechallenge with SARS-CoV-2. Moreover, passive transfer of convalescent serum to naïve hamsters efficiently suppressed the replication of the virus in the lungs even when the serum was administrated 2 d postinfection of the serum-treated hamsters. Collectively, these findings demonstrate that this Syrian hamster model will be useful for understanding SARS-CoV-2 pathogenesis and testing vaccines and antiviral drugs.

## Significance

Since SARS-CoV-2 emerged in China, it has spread rapidly around the world. Effective vaccines and therapeutics for SARS-CoV-2−induced disease (coronavirus disease 2019;COVID-19) are urgently needed. We found that SARS-CoV-2 isolates replicate efficiently in the lungs of Syrian hamsters and cause severe pathological lesions in the lungs of these animals similar to commonly reported imaging features of COVID-19 patients with pneumonia. SARS-CoV-2−infected hamsters mounted neutralizing antibody responses and were protected against rechallenge with SARS-CoV-2. Moreover, passive transfer of convalescent serum to naïve hamsters inhibited virus replication in their lungs. Syrian hamsters are a useful small animal model for the evaluation of vaccines, immunotherapies, and antiviral drugs.

The emergence of new pathogens in humans can impose huge public health and economic burdens on a global scale, in large part, because of lack of preexisting immunity. At the end of 2019, a novel coronavirus (severe acute respiratory syndrome coronavirus 2; SARS-CoV-2) that causes respiratory disease in and transmits among humans was detected in Wuhan, China (1, 2). On 11 March 2020, the World Health Organization declared that the infections caused by this new coronavirus had reached pandemic proportions.

A key strategy to protect humans from this coronavirus pandemic is the development of effective vaccines and therapeutics. Therefore, animal models that closely resemble the pathogenesis of SARS-CoV-2−induced disease, coronavirus disease 2019 (COVID-19), in humans are essential for research on disease mechanisms and for the evaluation of potential vaccines and antiviral drugs. While this manuscript was in preparation, Chan et al. (3) reported that SARS-CoV-2 caused a severe lung disease in hamsters and suggested that hamsters could serve as a useful mammalian model for COVID-19. These authors evaluated the pathogenicity and tissue tropism of SARS-CoV-2 isolates in hamsters after intranasal infection, and found that the virus replicated efficiently in the respiratory tract. They determined the infectious titer of the virus in only the nasal turbinates and lungs and did not examine the dissemination of SARS-CoV-2 isolates following intranasal inoculation, which therefore remains largely unknown.

Here, we analyzed the replicative ability of SARS-CoV-2 isolates by evaluating infectious titers in various organs in two different age groups of hamsters following inoculation by both the nasal and ocular routes. Chan et al. (3) showed that the virus replicated to higher titers in the upper respiratory tract (nasal turbinates) than in the lower respiratory tract (lungs). In contrast to their findings, we observed that the virus replicated efficiently in the respiratory tracts of both young and older infected animals, with no difference in its growth in the upper and lower respiratory tracts. By using in vivo X-ray microcomputed tomographic (micro-CT) imaging, we also examined the progression of lung inflammation caused by SARS-CoV-2 infection and the subsequent recovery processes in hamsters. Moreover, we assessed whether primary infection or passive transfer of convalescent serum could suppress the replication of SARS-CoV-2 in hamsters. In addition, by using scanning transmission electron microscopy (STEM) tomography, we observed the internal structure of SARS-CoV-2 virions.

## Results

### Growth Kinetics of SARS-CoV-2 Isolates in Mammalian Cells.

To characterize the new viruses, we examined the biological properties of two SARS-CoV-2 isolates: SARS-CoV-2/UT-NCGM02/Human/2020/Tokyo (UT-NCGM02) and SARS-CoV-2/UW-001/Human/2020/Wisconsin (UW-001), which were both isolated from mild cases. These isolates were propagated in VeroE6 or Vero 76 cells to prepare virus stocks. Titers of virus stocks were determined by use of plaque assays on the VeroE6 cell line VeroE6/TMPRSS2, which constitutively expresses transmembrane protease serine 2 (TMPRSS2), which activates SARS-CoV-2 infection (4).

**Fig. 1.**
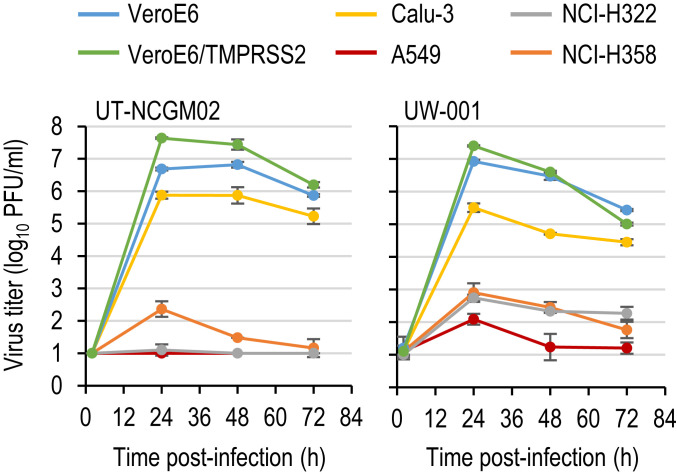
Growth kinetics of SARS-CoV-2 isolates in cell culture. VeroE6, VeroE6/TMPRSS2, Calu-3, A549, NCI-H322, and NCI-H358 cells were infected with viruses at an MOI of 0.05. The supernatants of the infected cells were harvested at the indicated times, and virus titers were determined by means of plaque assays in VeroE6/TMPRSS2 cells. Error bars indicate SDs from three independent experiments.

To identify cell lines capable of supporting the efficient replication of SARS-CoV-2 isolates, we first examined the growth kinetics of the two isolates in vitro (Fig. 1). Because the primary targets for SARS-CoV-2 are the epithelial cells in the airways and lungs in humans, the efficiencies of replication of the two isolates were compared in four human lung alveolar cell lines: Calu-3, A549, NCI-H322, and NCI-H358 cells. For comparison, we included VeroE6 and VeroE6/TMPRSS2 cells, which have previously been shown to support the efficient replication of SARS-CoV-2 (4, 5). These cells were infected with virus at a multiplicity of infection (MOI) of 0.05, and virus titers in the supernatant were measured by means of a plaque assay with VeroE6/TMPRSS2 cells. UT-NCGM02 grew efficiently in VeroE6 and VeroE6/TMPRSS2 cells, but it grew much faster and to higher titers (0.96 log units higher at 24 h postinfection) in the latter than in the former. UW-001 replicated with similar efficiency in VeroE6 and VeroE6/TMPRSS2 cells. Although the two isolates also grew in Calu-3 cells, the titers at 24 h postinfection were 0.81 to 1.89 log units lower than those in VeroE6 and VeroE6/TMPRSS2 cells, respectively. By contrast, these isolates did not show appreciable growth in A549, NCI-H322, or NCI-H358 cells. These findings are consistent with previous studies showing that VeroE6/TMPRSS2 and VeroE6 cells can efficiently support the replication of SARS-CoV-2 (4, 5). The entry of SARS-CoV-2 to target cells is initiated by the binding of its spike (S) glycoprotein to a receptor, human angiotensin-converting enzyme 2 (ACE2) (6, 7). A previous study showed that Vero and Calu-3 cells were more susceptible than A549 cells to virus entry driven by the S glycoprotein (6), suggesting that the expression of ACE2 may be relatively lower in A549 cells than in Vero and Calu-3 cells. Although ACE2 expression in NCI-H322 and NCI-H358 cells has not been examined, these cells may also express ACE2 at low levels.

### Three-Dimensional Structure of RNPs within SARS-CoV-2 Virions.

**Fig. 2.**
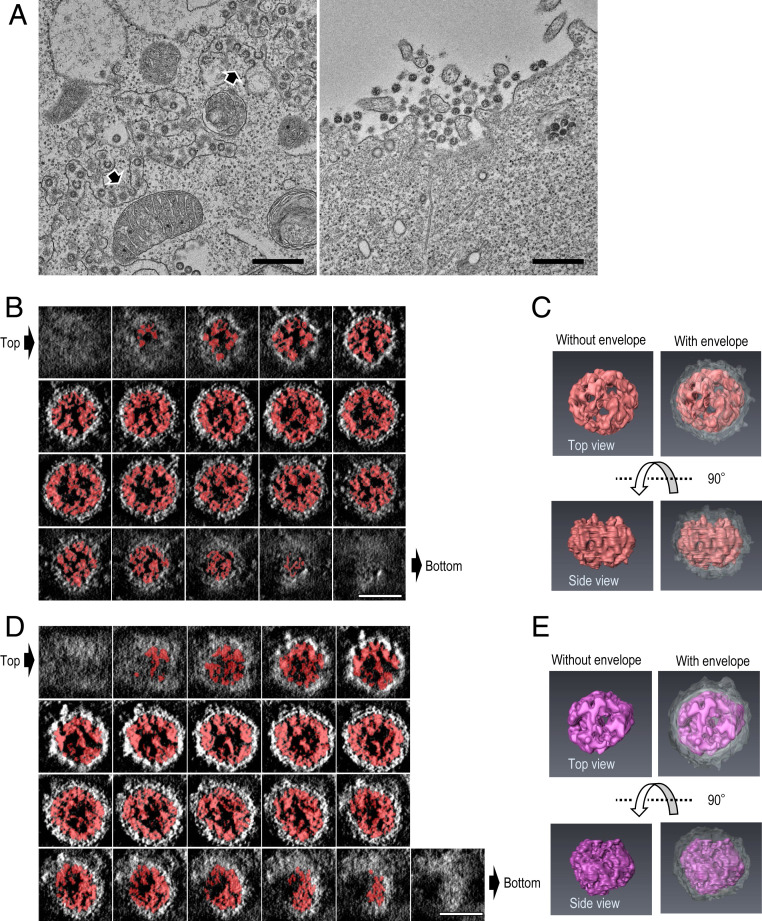
TEM and STEM tomography images of SARS-CoV-2 virions in infected VeroE6/TMPRSS2 cells. VeroE6/TMPRSS2 cells were infected with UT-NCGM02 at an MOI of 1. At 22 h postinfection, the infected cells were fixed, and analyzed using thin-section TEM and STEM tomography. (*A*) TEM images of virions. Virions were seen within intracellular compartments (*Left*) and in the extracellular space adjacent to the plasma membrane (*Right*). Arrow heads indicate virions budding into the intracellular compartments. (Scale bar, 500 nm.) (*B*–*E*) STEM tomography images of virions. Semithin sections (250 nm thick) were prepared from the same samples as those examined by using TEM (in *A*). Then, 3D structures of the whole virions were computationally reconstructed by using STEM tomography. (*B* and *D*) Consecutive transverse sections (0.72 nm thick at 1.4-nm intervals) of the reconstructed virions are shown from the top (top left) to the bottom (bottom left). (Scale bar, 50 nm.) (*C* and *E*) The 3D models of the RNPs (red and purple) within the virions from the top (*Right*) and side (*Left*) views. The viral envelope is colored gray.

Assembly and budding of progeny coronavirus virions occur in tubular structures between the endoplasmic reticulum (ER) and the Golgi complex (also known as the ER−Golgi intermediate compartment) (8, 9). Transmission EM revealed that numerous virions bud inside the tubular/vesicular structures in virus-infected VeroE6/TMPRSS2 cells. These virions (*n* = 61) had a diameter of 81 nm to 125 nm, which is consistent with previous EM observations (Fig. 2*A*) (2). A large number of extracellular virions were also observed on the cell surface.

Coronaviruses possess helical ribonucleoprotein complexes (RNPs) that consist of a nucleoprotein and a positive-sense, single-stranded RNA genome. We examined thin sections (∼250 nm thick) of virus-infected cells by using STEM tomography to elucidate the three-dimensional (3D) structure of the RNPs within these virions (Fig. 2 *B*–*E* and Movies S1 and S2). STEM tomography allows 3D imaging of an entire virion and its internal components. Preliminary analysis of our STEM tomography images revealed that the RNPs within the virion are arranged beneath the envelope as irregular helical structures. We also observed that the RNPs of SARS-CoV-2 appear to associate with the inner face of the lipid envelope, as reported for SARS-CoV-1 (10). Although we analyzed a limited number of virions, the information we obtained about the structures inside SARS-CoV-2 is informative. Follow-up studies are needed to reveal the detailed 3D structures of the RNPs of SARS-CoV-2.

### Pathogenicity and Replication of SARS-CoV-2 in Hamsters.

**Fig. 3.**
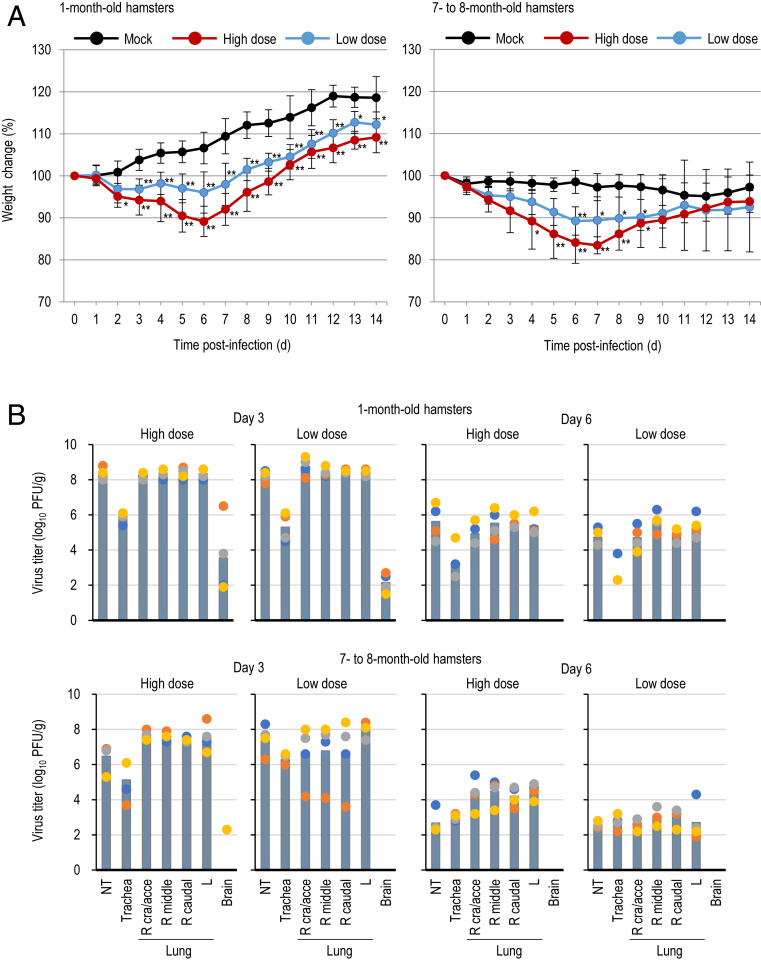
Virus replication in infected Syrian hamsters. (*A*) Body weight changes in Syrian hamsters after viral infection. Syrian hamsters were inoculated with 10^5.6^ PFU (in 110 μL) or with 10^3^ PFU (in 110 μL) of UT-NCGM02 or PBS (mock) via a combination of the intranasal (100 μL) and ocular (10 μL) routes. Body weights of virus-infected (*n* = 4) and mock-infected hamsters (*n* = 4) were monitored daily for 14 d. Data are presented as the mean percentages of the starting weight (±SD). *P* values were calculated by using pairwise comparisons after a linear mixed model analysis (*P < 0.05; **P < 0.01). Asterisks next to data points indicate statistically significant differences between virus- and mock-infected animals. See *Methods* for more details regarding the statistical analysis. (*B*) Virus replication in infected Syrian hamsters. Syrian hamsters were inoculated with 10^5.6^ PFU (in 110 μL) or with 10^3^ PFU (in 110 μL) of UT-NCGM02 via a combination of the intranasal (100 μL) and ocular (10 μL) routes. Four Syrian hamsters per group were killed on days 3, 6, and 10 postinfection for virus titration. Virus titers in various organs were determined by means of plaque assays in VeroE6/TMPRSS2 cells. Vertical bars show the mean. The vertical bar is shown only when virus was recovered from all four hamsters. Points indicate data from individual Syrian hamsters. NT, nasal turbinate; R cra/acce, right cranial and accessory lobes; R middle, right middle lobe; R caudal, right caudal lobe; L, left lobe.

SARS-CoV-2 is closely related genetically to the SARS-CoV-1 that emerged in 2003. Given that Syrian hamsters are susceptible to SARS-CoV-1 (11–13), we evaluated the replication and pathogenicity of SARS-CoV-2 in these hamsters. Epidemiologic data indicate that the elderly are more prone to severe outcomes with COVID-19 than younger individuals (14, 15), suggesting that the age of the host may influence the pathogenesis of SARS-CoV-2. We used two age groups of Syrian hamsters: 1 mo old (juvenile) and 7 mo to 8 mo old (mature adults). Hamsters were infected with 10^5.6^ plaque-forming units (PFU) or with 10^3^ PFU of UT-NCGM02 through a combination of the intranasal and ocular routes; the ocular inoculation route was included because conjunctivitis has been reported among COVID-19 patients (16). In the younger age group, the body weight of mock-infected hamsters had gradually increased by 14 d postinfection (Fig. 3*A* and *SI Appendix*, Fig. S1). In contrast, of the animals infected with the low dose, three showed slight weight loss by day 6 postinfection (6.1 to 8.8%). Although the remaining one hamster did not exhibit weight loss, it gained weight more slowly than the mock-infected animals. All four animals infected with the high dose exhibited weight loss (range, 7.9 to 15.4%) by day 6 postinfection. By day 14 postinfection, all eight animals infected with either dose had gained less weight than the mock-infected animals. In the older age groups, mock-infected Syrian hamsters exhibited little to no weight loss (<7.8%). Although three of the four animals infected with the low dose showed only modest weight loss by day 7 postinfection (8.9 to 10.4%), the remaining hamster exhibited severe weight loss at this time point (18.5%) and continued to lose weight for up to 14 d postinfection (23.3%). All four animals infected with the high dose experienced substantial weight loss by day 7 postinfection (13.8 to 21.9%).

We next investigated the replicative ability of UT-NCGM02 in Syrian hamsters (Fig. 3*B* and *SI Appendix*, Tables S1 and S2). In both age groups, infection of hamsters with UT-NCGM02 resulted in high virus titers in the nasal turbinates, trachea, and lungs, with no appreciable difference between the inoculation doses at day 3 postinfection. Viruses were also recovered from the brains of all eight animals infected with the high or low dose in the younger age group (note that the brain samples collected for virus titration included the olfactory bulb). On day 6 postinfection, virus was detected in the respiratory organs of animals infected with the high or low dose in both the younger and older age groups, but was not detected in any other organs tested. Virus titers in the respiratory organs of the infected animals were lower on day 6 than on day 3 postinfection. No substantial difference in viral titers in the respiratory organs on days 3 and 6 postinfection was observed between the two age groups. At day 10 postinfection, no virus was recovered from the organs of almost all of the infected animals, with the exception of the trachea of one of the four animals infected with the high dose and the nasal turbinates of one of the four animals infected with the low dose in the younger age group.

### Micro-CT Imaging of the Lungs of SARS-CoV-2−Infected Hamsters.

**Fig. 4.**
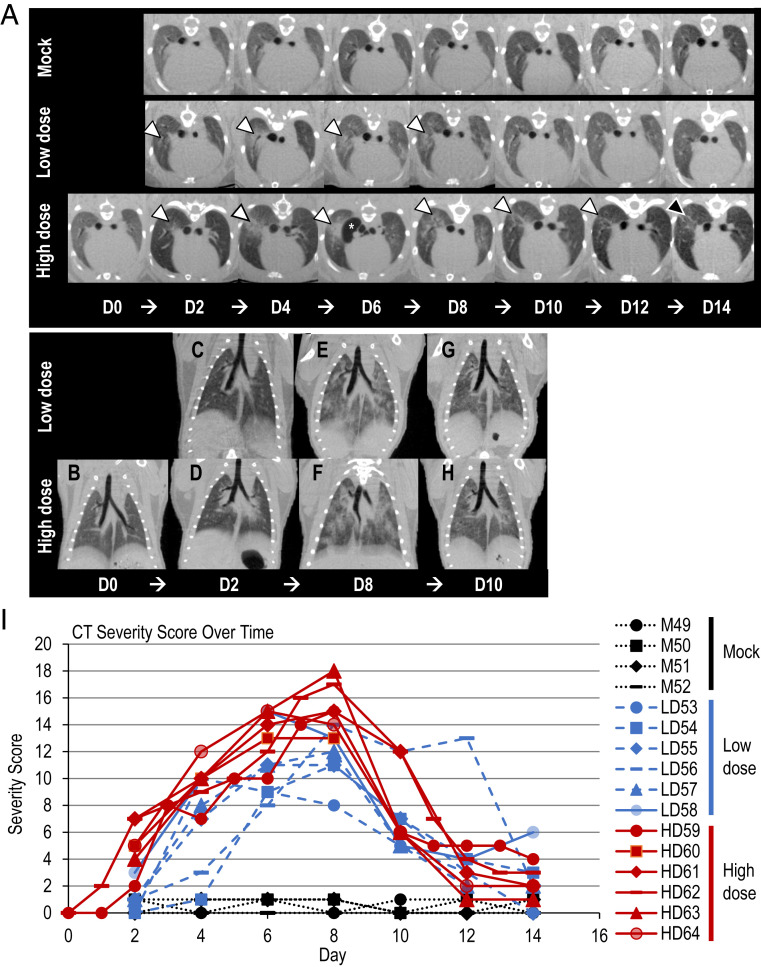
Micro-CT imaging of the lungs of infected Syrian hamsters. (*A*) Axial CT images of the thorax in mock-infected control, low dose-infected, and high dose-infected animals showing lung abnormalities over a 14-d period (white arrowheads). Lung abnormalities were first detected 2 d postinfection, and the most severe changes were observed 8 d postinfection in virus-infected animals. The high dose-infected animals had, overall, more severe lung abnormalities compared to the low dose-infected animals. Lung abnormalities began to improve 10 d postinfection for both low dose- and high dose-infected animals. On day 14 postinfection, the high dose-infected animals had a higher degree of residual lung abnormalities compared to the low dose-infected animals, highlighted by the black arrowhead. Pneumomediastinum is labeled by the white asterisk (*). Note that the day 0 control image was only obtained for the high dose-infected animal and is not available for the low dose-infected or mock-infected animals. (*B*–*H*) Dorsal/coronal plane reconstruction CT images of the thorax in low dose-infected and high dose-infected animals showing (*B*) a control image, (*C* and *D*) initial lung changes, (*E* and *F*) most severe lung changes, and (*G* and *H*) the beginning of the recovery phase over time. The high dose-infected animals had more severe lung abnormalities than the low dose-infected animals. Note that the day 0 control image was only obtained for the high dose-infected animals and is not available for the low dose-infected animals. (*I*) CT severity score of mock-infected control and infected Syrian hamsters. CT Severity Score over time for mock-infected control, low dose-infected, and high dose-infected animals over 14 d. Low dose- and high dose-infected animals had severe lung abnormalities as demonstrated by high CT severity scores compared to mock-infected control animals. High dose-infected animals had a higher CT severity score compared to low dose-infected animals. CT abnormalities were first detected 2 d postinfection, the most severe changes peaked at 6 d to 8 d postinfection, and the recovery period began 10 d postinfection. Mild lung abnormalities, as indicated by lower CT severity scores, persisted at 14 d postinfection in most of the infected animals.

Because no substantial difference in viral titers in the respiratory organs was observed between the two age groups, we performed qualitative and semiquantitative image analysis of the lung abnormalities only in the younger age group after SARS-CoV-2 infection, by using a CT severity score adapted from a human scoring system (17). The micro*-*CT analysis revealed severe lung abnormalities in all infected animals that were not present in the mock-infected control animals (Fig. 4 and Movies S3–S7). CT lung abnormalities were first detected at day 2 postinfection (range 2 d to 4 d, mean 2.5 d) and began as ill-defined, patchy ground glass opacity (GGO) with a central, peribronchial distribution. Lung abnormalities then progressed to more severe, peripherally distributed, rounded, multilobular GGO with regions of lung consolidation. The right cranial lung lobe was most commonly affected first, and lung abnormalities were more severe in right lung lobes compared to left throughout the study period (Fig. 4 *A*–*H*). The most severe lung changes occurred 7 d to 8 d postinfection (range 6 d to 8 d, mean 7.17 d), and correlated with the highest CT severity scores (Fig. 4*I*). High dose-infected animals had more severe lung abnormalities (CT severity score ranging from 0 to 18 [mean 8.62, median 8.50]) than low dose-infected animals (CT severity score ranging from 0 to 15 [mean 6.23, median 5.79]). All infected animals developed a pneumomediastinum 4 d to 6 d postinfection (mean 5.42 d) that resolved by 8 d to 10 d postinfection (mean 8.5 d). This finding was unexpected and likely secondary to severe pulmonary damage, micropulmonary rupture, and gas tracking into the mediastinum. High dose-infected animals developed a pneumomediastinum slightly earlier (mean 5.17 d) than low dose-infected animals (mean 6 d).

Improvement of CT lung abnormalities began 8 d to 10 d postinfection (range 8 d to 10 d, mean 9.45 d), with gradual decrease in GGO and lung remodeling evident by linear soft tissue bands at sites of lung injury. Residual, minimal lung abnormalities/remodeling including ill-defined GGO and linear bands remained in 10 of 11 infected animals imaged on day 14 postinfection, and in 6 of 10 infected animals imaged on day 20 postinfection. A higher percentage of high dose-infected animals (75%, 3/4) than low dose-infected animals (50%, 3/6) had minimal residual lung abnormalities on the final CT scan on day 20.

### Histopathological Examination of the Lungs of SARS-CoV-2−Infected Hamsters.

**Fig. 5.**
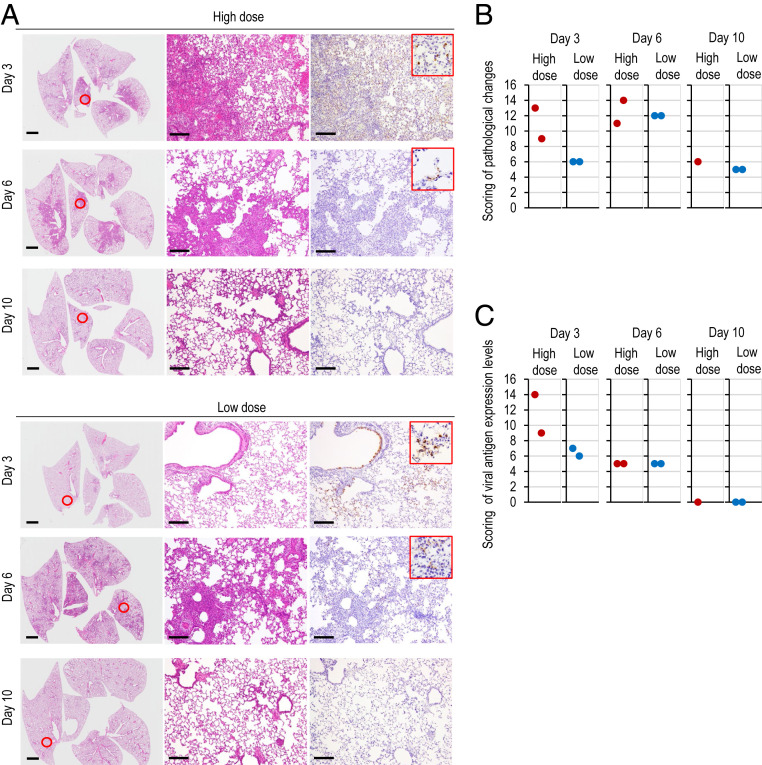
Pathological findings in infected Syrian hamsters. (*A*) Histopathological examination of the lungs of infected hamsters. Syrian hamsters were inoculated with 10^5.6^ PFU (in 110 μL) or with 10^3^ PFU (in 110 μL) of UT-NCGM02 via a combination of the intranasal (100 μL) and ocular (10 μL) routes. Syrian hamsters infected with the high or low dose were killed on days 3, 6, and 10 postinfection for pathological examinations (*n* = 2, except for 1 in the high-dose group on day 10). Shown are representative pathological findings in the lungs of hamsters infected with the virus on days 3, 6, and 10 postinfection (*Left* and *Middle*, hematoxylin and eosin staining; *Right*, immunohistochemistry for SARS-CoV-2 antigen detection). *Middle* and *Right* show enlarged views of the area circled in red in *Left*. (Scale bars, 2 mm [*Left*] and 200 μm [*Middle* and *Right*].) (*B* and *C*) Pathological severity scores in infected hamsters. To evaluate comprehensive histological changes, lung tissue sections were scored based on (*B*) pathological changes and (*C*) viral antigen detection levels. (*B*) Scores were determined based on the percentage of inflammation area for each section of the five lobes collected from each animal in each group by using the following scoring system: 0, no pathological change; 1, affected area (≤10%); 2, affected area (<50%, >10%); 3, affected area (≥50%); an additional point was added when pulmonary edema and/or alveolar hemorrhage was observed. The total score for the five lobes is shown for individual animals. (*C*) Scores were also determined based on the percentage of virus antigen-positive cells, as determined by immunohistochemistry, for each section of the five lobes collected from each animal in each group by using the following scoring system: 0, no positive cells; 1, positive cells (≤10%); 2, positive cells (<50%, >10%); 3, positive cells (≥50%). The total score for the five lobes is shown for individual animals.

We further examined the histopathological changes in the respiratory organs of the younger age group after SARS-CoV-2 infection (Fig. 5). Pathological examination of hamsters infected with UT-NCGM02 revealed severe lung lesions on day 3 postinfection that extended across larger areas for hamsters infected with the high dose compared with those infected with the low dose (Fig. 5 *A* and *B*), consistent with our micro-CT observations that the animals infected with the high dose developed a pneumomediastinum earlier than those infected with the low dose (Fig. 4 *A*–*H*). At 6 d postinfection, there were no differences in the histological changes between the lungs of the animals infected with the low dose and the lungs of the animals infected with the high dose (Fig. 5 *A* and *B*). Focal inflammatory cell infiltration in the interstitium and the alveolar cavity were prominent, and pulmonary edema and alveolar hemorrhage were evident in some areas of the lungs of animals infected with the high dose on day 3 postinfection and in the lungs of animals infected with either dose on day 6 postinfection. Viral antigen-positive cells were detected in the bronchi and/or lungs of all eight animals infected with either dose on days 3 and 6 postinfection; however, more virus antigen-positive cells were detected in the lungs of virus-infected animals on day 3 than on day 6 postinfection (Fig. 5 *A* and *C*), which is consistent with the results of virus titration (Fig. 3*B*). Virus antigens were also observed in the nasal mucosa and/or olfactory epithelium of all eight animals infected with either dose on days 3 and 6 postinfection (*SI Appendix*, Fig. S2*A*). Our virological data revealed that SARS-CoV-2 may enter the brain of infected animals (Fig. 3*B*); however, viral antigens were not detected in the brain sections of the animals tested. Therefore, it is unclear whether SARS-CoV-2 can infect the brain. Of the animals infected with the high dose, one was humanely killed on day 7 postinfection because it had lost more than 25% of its initial body weight at this time point. Pathological examination of the lung showed a few virus-positive cells and severe inflammation (*SI Appendix*, Fig. S2*B*). By contrast, at 10 d postinfection, pathological changes were slight, and no viral antigen-positive cells were detected in the lungs of animals infected with either dose. This result was consistent with that of our micro*-*CT analysis in which improvement of CT lung abnormalities began 8 d to 10 d postinfection (Fig. 4*I*).

### Rechallenge with SARS-CoV-2.

We next asked whether hamsters that developed antibodies against SARS-CoV-2 were resistant to subsequent reinfection. Six hamsters that had previously received the high or low dose of UT-NCGM02 were rechallenged with 10^5.6^ PFU of homologous virus on day 20 after the primary infection. At day 4 after infection, in the mock-infected control group, high virus titers were detected in the respiratory tract (Table 1). In contrast, no virus was isolated from any of the respiratory organs of all six animals that were previously infected and then reinfected with the virus. Enzyme-linked immunosorbent and virus neutralization assays with sera collected on day 19 after the primary infection revealed that all of the infected animals had seroconverted (Table 1). These observations indicate that primary SARS-CoV-2 infection provides protective immunity against subsequent reinfection.

**Table 1.** Virus replication in the respiratory tract of hamsters following rechallenge

Primary infection dose	Animal ID	Antibody endpoint titer in serum*	Neutralizing antibody titer in serum^†^	Virus titers (log_10_ PFU/g) of animals infected with SARS-CoV-2^‡^		
				Nasal turbinate	Trachea	Lung
High dose (10^5.6^ PFU)	#1	40,960	1,280	—	—	—
	#2	40,960	640	—	—	—
	#3	40,960	1,280	—	—	—
Low dose (10^3^ PFU)	#4	40,960	640	—	—	—
	#5	40,960	640	—	—	—
	#6	40,960	1,280	—	—	—
Mock (PBS)	#7	<10	<20	5.5	3.3	7.1
	#8	<10	<20	5.4	4.7	7.4
	#9	<10	<20	5.3	3.5	7.4

Syrian hamsters were inoculated with 10^5.6^ PFU (in 110 μL) of UT-NCGM02 via a combination of the intranasal (100 μL) and ocular (10 μL) routes on day 20 after the primary infection. Three Syrian hamsters per group were killed on day 4 after rechallenge for virus titration.

* Viral antibody endpoint titers against the receptor-binding domain expressed as the reciprocal of the highest dilution with an optical density at 490 nm (OD_490_) cutoff value >0.15; sera were collected on day 19 after the primary infection.

^†^ Viral neutralization titers against inoculated virus; sera were collected on day 19 after the primary infection.

^‡^ A dash denotes that virus was not detected.

### Inhibitory Effects of Serum Antibodies on the Replication of SARS-CoV-2.

We next assessed the protectivity of convalescent serum from infected animals on the replication of SARS-CoV-2 in the respiratory tracts of hamsters. Postinfection sera were collected from hamsters that had been infected with the high or low dose of UT-NCGM02 and then pooled. The pooled serum was then transferred intraperitoneally to three hamsters on day 1 or 2 after infection with 10^3^ PFU of the virus. Normal uninfected hamster serum was injected intraperitoneally into three naïve hamsters as a control. Virus titers in the nasal turbinates and lungs of the animals that received postinfection serum on day 1 postinfection were statistically significantly lower than the virus titers in those organs of animals that received normal serum at the corresponding time point postinfection (*P* < 0.05 and *P* < 0.01 for the virus titers in the nasal turbinates and lungs, respectively) (Table 2 and *SI Appendix*, Table S3). Although no statistically significant differences in the virus titers in the respiratory organs were found between the animals that received postinfection serum and those that received normal serum on day 2 postinfection, the virus titers in the lungs of animals that received postinfection serum were appreciably lower (5.9 ± 1.8 log10 PFU ± SD/g) than those in the lungs of animals that received normal serum (7.8 ± 0.1 PFU ± SD/g). We found that, of the animals that received postinfection serum, only animal #9, which received postinfection serum on day 2 after infection, had a lung virus titer similar to that of animals that received normal serum. This animal was the only animal that had undetectable levels of antibody to SARS-CoV-2, indicating that the serum was not successfully administered to this animal (*SI Appendix*, Table S3). We also found that viral replication was more effectively prevented in the lungs than in the nasal turbinates. Our findings are consistent with previous reports that passive transfer of immune serum to mice or hamsters prevents the replication of SARS-CoV-1 or SARS-CoV-2, respectively, in the lungs (3, 18). Taken together, these results indicate that the passive transfer of convalescent serum from infected animals could restrict viral replication in the respiratory tract of infected animals even if the serum is administrated after infection has occurred.

**Table 2.** Effect of convalescent serum on the replication of SARS-CoV-2 in hamsters

Serum was administered to recipient hamsters on:	Passively transferred serum	Neutralizing antibody titer in serum*	Virus titers (mean log_10_ PFU ±SD/g) in:	
			Nasal turbinate^†^	Lung^‡^
Day 1 postinfection	Infected hamster serum	640	6.1 ± 0.4	5.3 ± 0.4
	Uninfected hamster serum	<10	7.4 ± 0.3	8.2 ± 0
Day 2 postinfection	Infected hamster serum	640	6.3 ± 0.7	5.9 ± 1.8
	Uninfected hamster serum	<10	6.7 ± 0.3	7.8 ± 0.1

Syrian hamsters (*n* = 3 for each group) were inoculated intranasally with 10^3^ PFU of UT-NCGM02. On day 1 or 2 postinfection, the hamsters were injected intraperitoneally with postinfection serum or normal uninfected serum. Animals were killed on day 4 postinfection for virus titration.

* Viral neutralization titers against inoculated virus; sera were collected from eight hamsters on day 38 or 39 postinfection and then pooled.

^†^ Statistical significance was calculated by using two-tailed unpaired Student’s *t* tests; the *P* value was <0.05 compared with the virus titers in the nasal turbinates of hamsters that received serum from uninfected hamsters.

^‡^ Statistical significance was calculated by using two-tailed unpaired Student’s *t* tests; the *P* value was <0.01 compared with the virus titers in the lungs of hamsters that received serum from uninfected hamsters.

## Discussion

In COVID-19 patients with acute respiratory illness, the main clinical manifestation is severe lung inflammation. Consistent with a previous study using Syrian hamsters (3), our data demonstrated that SARS-CoV-2 replicates efficiently in the lungs of Syrian hamsters and causes severe pathological lesions in the lungs of these animals following SARS-CoV-2 infection. In addition, our micro*-*CT analysis revealed that severe lung injury occurs in infected hamsters and that the severity of the lung abnormalities is related to the degree of infectious dose. Commonly reported imaging features of COVID-19 patients with pneumonia (19) were present in all infected animals but not in mock-infected control animals. These findings indicate that the pathological features of the lungs of SARS-CoV-2−infected hamsters resemble those observed in COVID-19 patients (17, 19–21). The observed trends of CT lung changes in infected hamsters over time may provide valuable clinical insight into SARS-CoV-2 infection and recovery. Computational modeling suggests that ACE2 from Chinese hamster could interact with the S glycoprotein of SARS-CoV-2 (22). Thus, this animal would be a valuable model to improve our understanding of the pathogenesis of lung injury caused by SARS-CoV-2 infection. In addition, Chan et al. (3) demonstrated that this hamster model is a useful tool for studies on SARS-CoV-2 transmission.

We observed that the virus titers in the nasal turbinates and lungs of infected Syrian hamsters did not correlate with the dose of virus administered (Fig. 3*B*). Similar findings were obtained with Syrian hamsters infected with SARS-CoV-1; the viral titers in the lungs following intranasal administration of 10^3^ or 10^5^ 50% tissue culture infective doses (TCID_50_) of SARS CoV-1 were similar on day 3 postinfection regardless of the dose administered (13). Thus, in the hamster model, SARS-CoV-1 and SARS-CoV-2 can replicate to high titers in the respiratory organs, even when the virus is administered at a low dose. However, histopathology and body weight changes differ depending on the dose of virus administered; therefore, these parameters can be used to evaluate virus pathogenicity and the effectiveness of countermeasures when a high dose of virus is used to infect hamsters.

Chan et al. (3) showed that SARS-CoV-2 replicates to higher titers in the nasal turbinates than in the lungs of infected Syrian hamsters. In contrast, we observed that the level of virus replication in the lungs was comparable to that in the nasal turbinates (Fig. 3*B*). Chan et al. (3) and we used almost identical experimental designs, with no substantial differences in hamster age, gender, inoculation dose, or volume. Therefore, the reason for this discrepancy is unclear. However, it might be due to differences in the virus preparations; Chen et al. (3) used plaque-purified viruses amplified in VeroE6 cells, whereas we used isolates amplified in the same cells without plaque purification. The presence of quasispecies in patient samples has been documented for SARS-CoV-1 and SARS-CoV-2 isolates (23, 24). Therefore, it is possible that plaque purification may have resulted in the selection of viruses with reduced replication fitness in the lungs of hamsters.

Our data show that SARS-CoV-2 can replicate in the brain or olfactory bulb of animals (Fig. 3*B*); however, we detected viral antigens in neither the brain nor the olfactory bulb of infected hamsters. Previous studies have reported that coronaviruses, such as SARS-CoV-1, and mouse hepatitis viruses could enter the central nervous system following intranasal inoculation of mice (25, 26). Interestingly, acute olfactory impairment (anosmia) has been recognized as an early symptom of COVID-19 patients (27), suggesting that the virus may infect cells within the olfactory epithelium of humans, as we detected in hamsters (*SI Appendix*, Fig. S2*A*). Further investigations are required to determine whether SARS-CoV-2 isolates could spread from the nasal cavity to the nervous system via the olfactory route.

Our studies demonstrated that primary SARS-CoV-2 infection elicited neutralizing antibodies that protected hamsters from subsequent infection. Similar findings have been reported in reinfection experiments of SARS-CoV-1 using mice (18). These data support the concept that people who recover from COVID-19 would be protected from reinfection at least for a period of time while their immunity to SARS-CoV-2 lasted. These results serve as a rationale for the development of live attenuated SARS-CoV-2 vaccines and other vaccines that induce protective antibodies.

We also showed the protective effects of convalescent serum in our hamster model of SARS-CoV-2 infection (Table 2 and *SI Appendix*, Table S3); the levels of virus titer reduction were substantial in animals treated with convalescent sera (i.e., nearly 1,000-fold virus titer reduction in the lungs of animals inoculated with convalescent sera 1 d postinfection and nearly 100-fold virus titer reduction in those inoculated on day 2 postinfection). Chan et al. (3) also reported a protective effect of convalescent serum in their hamster model, although the level of protection was not substantial for reasons that are currently unclear. Nonetheless, both datasets suggest that anti−SARS-CoV-2 polyclonal hyperimmune globulin from convalescent sera from COVID-19 patients and monoclonal antibodies to SARS-CoV-2 could reduce viral load in patients.

In conclusion, our data indicate that hamsters are highly susceptible to infection with SARS-CoV-2, without the need for prior adaptation, and develop severe pneumonia similar to COVID-19 patients. Importantly, the use of this animal model would facilitate the rapid evaluation of vaccine or antiviral therapy candidates at a relatively low cost compared to other animal models such as ferrets and nonhuman primates, which have also been shown to be susceptible to SARS-CoV-2 infection (28, 29). In addition, the vast majority of hamsters did not die upon SARS-CoV-2 infection, which is consistent with human infections. It would be interesting and important to develop COVID-19 hamster models with comorbidities such as diabetes mellitus, hypertension, or obesity (30). Taken together, our findings demonstrate that this Syrian hamster model will be useful for understanding SARS-CoV-2 pathogenesis and testing vaccines and antiviral drugs.

## Methods

### Viruses.

SARS-CoV-2 isolates were propagated in VeroE6 cells in Opti-MEM I (Invitrogen) containing 0.3% bovine serum albumin (BSA) and 1 µg of l-1*-*tosylamide*-*2*-*phenylethyl chloromethyl ketone treated-trypsin per mL or in Vero 76 cells in Eagle’s minimal essential medium (MEM) supplemented with 2% fetal calf serum at 37 °C.

All experiments with SARS-CoV-2 were performed in enhanced biosafety level 3 (BSL3) containment laboratories at the University of Tokyo, which are approved for such use by the Ministry of Agriculture, Forestry, and Fisheries, Japan, or in enhanced BSL3 containment laboratories at the University of Wisconsin-Madison, which are approved for such use by the Centers for Disease Control and Prevention and by the US Department of Agriculture.

### Experimental Infection of Syrian Hamsters.

One-month-old female Syrian hamsters (Japan SLC Inc.) and 7- to 8-mo-old female Syrian hamsters (Envigo) were used in this study. Baseline body weights were measured before infection. Under ketamine−xylazine anesthesia, four hamsters per group were inoculated with 10^5.6^ PFU (in 110 μL) or with 10^3^ PFU (in 110 μL) of UT-NCGM02 via a combination of the intranasal (100 μL) and ocular (10 μL) routes. Body weight was monitored daily for 14 d.

For virological and pathological examinations, two, four, or five hamsters per group were infected with 10^5.6^ PFU (in 110 μL) or with 10^3^ PFU (in 110 μL) of the virus via a combination of the intranasal and ocular routes; 3, 6, and 10 d postinfection, the animals were killed, and their organs (nasal turbinates, trachea, lungs, eyelids, brain, heart, liver, spleen, kidneys, jejunum, colon, and blood) were collected.

For the reinfection experiments, three hamsters per group were infected with 10^5.6^ PFU (in 110 μL) or with 10^3^ PFU (in 110 μL) of UT-NCGM02 or PBS (mock) via a combination of the intranasal and ocular routes. On day 20 postinfection, these animals were reinfected with 10^5.6^ PFU of the virus via a combination of the intranasal and ocular routes. On day 4 after reinfection, the animals were killed, and the virus titers in the nasal turbinates, trachea, and lungs were determined by means of plaque assays in VeroE6/TMPRSS2 cells.

For the passive transfer experiments, eight hamsters were infected with 10^5.6^ PFU (in 110 μL) or with 10^3^ PFU (in 110 μL) of UT-NCGM02 via a combination of the intranasal and ocular routes. Serum samples were collected from these infected hamsters on day 38 or 39 postinfection, and were pooled. Control serum was obtained from uninfected age-matched hamsters. Three hamsters per group were inoculated intranasally with 10^3^ PFU of UT-NCGM02. On day 1 or 2 postinfection, hamsters were injected intraperitoneally with the postinfection serum or control serum (2 mL per hamster). The animals were killed on day 4 postinfection, and the virus titers in the nasal turbinates and lungs were determined by means of plaque assays in VeroE6/TMPRSS2 cells. All experiments with hamsters were performed in accordance with the Science Council of Japan’s Guidelines for Proper Conduct of Animal Experiments and the guidelines set by the Institutional Animal Care and Use Committee at the University of Wisconsin–Madison. The protocol was approved by the Animal Experiment Committee of the Institute of Medical Science, the University of Tokyo (approval no. PA19-75) and the Animal Care and Use Committee of the University of Wisconsin–Madison (protocol no. V00806).

Detailed materials and methods for this study are described in *SI Appendix*.

### Date Availability.

All data supporting the findings of this study are included in the main text and *SI Appendix*; any materials will be made available upon request.

## Supplementary Material

Supplementary File

Supplementary File

Supplementary File

Supplementary File

Supplementary File

Supplementary File

Supplementary File

Supplementary File
