# Supplementary material for: Syrian hamsters as a small animal model for SARS-CoV-2 infection and countermeasure development
Source: Proc Natl Acad Sci U S A. 2020 Jun 22;117(28):16587–95. doi: 10.1073/pnas.2009799117 (PMC7368255; doi:10.1073/pnas.2009799117)
Supplement: Supplementary File [file pnas.2009799117.sapp.pdf]

PNAS
www.pnas.org

Supplementary Information for

Syrian hamsters as a small animal model for SARS-CoV-2 infection and countermeasure development

Masaki Imai[1,9], Kiyoko Iwatsuki-Horimoto[1,9], Masato Hatta[2,9], Samantha Loeber[3,9], Peter J. Halfmann[2,9], Noriko Nakajima[4,9], Tokiko Watanabe[1], Michiko Ujie[1], Kenta Takahashi[4], Mutsumi Ito[1], Shinya Yamada[1], Shufang Fan[2], Shiho Chiba[2], Makoto Kuroda[2], Lizheng Guan[2], Kosuke Takada[1], Tammy Armbrust[2], Aaron Balogh[2], Yuri Furusawa[1], Moe Okuda[1], Hiroshi Ueki[1], Atsuhiro Yasuhara[1], Yuko Sakai-Tagawa[1], Tiago J. S. Lopes[1,2], Maki Kiso[1], Seiya Yamayoshi[1], Noriko Kinoshita[5], Norio Ohmagari[5], Shin-ichiro Hattori[5], Makoto Takeda[6], Hiroaki Mitsuya[5], Florian Krammer[7], Tadaki Suzuki[4], Yoshihiro Kawaoka[1,2,8]

[1]*Division of Virology, Department of Microbiology and Immunology, Institute of Medical Science, University of Tokyo, Tokyo 108-8639, Japan*
[2]*Influenza Research Institute, Department of Pathobiological Sciences, School of Veterinary Medicine, University of Wisconsin-Madison, WI 53711, USA*
[3]*Department of Surgical Sciences, School of Veterinary Medicine, University of Wisconsin-Madison, WI 53706, USA*
[4]*Department of Pathology, National Institute of Infectious Diseases, Tokyo 162-8640, Japan*
[5]*Disease Control and Prevention Center, National Center for Global Health and Medicine, Tokyo 162-8655, Japan*
[6]*Department of Virology 3, National Institute of Infectious Diseases, Musashimurayama, Tokyo 208-0011, Japan*
[7]*Department of Microbiology, Icahn School of Medicine at Mount Sinai, New York, NY 10029, USA*
[8]*Department of Special Pathogens, International Research Center for Infectious Diseases, Institute of Medical Science, University of Tokyo, Minato-ku, Tokyo 108-8639, Japan*
[9]*These authors contributed equally*

Yoshihiro Kawaoka
Email: yoshihiro.kawaoka@wisc.edu

This PDF file includes:

Supplementary methods
Figures S1 to S2
Tables S1 to S3
Legends for Movies S1 to S7
SI References

Other supplementary materials for this manuscript include the following:

Movies S1 to S7

Supplementary methods

Cells. VeroE6 (ATCC CRL-1586), Calu-3 (ATCC HTB-55), and Vero 76 (ATCC CRL-1587) cells were maintained in Eagle's minimal essential media (MEM) containing 10% fetal calf serum (FCS) and antibiotics. Human lung carcinoma epithelial A549 cells were propagated in Ham's F12K Nutrient Medium containing 10% FCS and antibiotics. NCI-H322 (ATCC CRL-5806) and NCI-H358 (ATCC CRL-5807) cells were maintained in Roswell Park Memorial Institute 1640 medium (RPMI 1640) containing 10% FCS and antibiotics. VeroE6/TMPRSS2 (1) (JCRB 1819) cells were propagated in the presence of 1 mg/ml geneticin (G418; Invivogen) and 5 µg/ml plasmocin prophylactic (Invivogen) in Dulbecco's Modified Eagle Medium (DMEM) containing 10% FCS and antibiotics. All cells were incubated at 37 °C with 5% CO_2, and regularly tested for mycoplasma contamination by using PCR and were confirmed to be mycoplasma-free.

Clinical specimens. Respiratory swabs were obtained from a patient with laboratory-confirmed COVID-19, who was hospitalized at the Center Hospital of the National Center for Global Health and Medicine, Tokyo, Japan. The swabs were submitted to the Division of Virology, Department of Microbiology and Immunology, Institute of Medical Science, the University of Tokyo for virus isolation by inoculating with VeroE6 cells. The research protocol was approved by the Research Ethics Review Committee of the Institute of Medical Science of the University of Tokyo.

A nasopharyngeal specimen was collected from an individual with a laboratory-confirmed case of COVID-19 and submitted to the Influenza Research Institute, Department of Pathobiological Sciences, School of Veterinary Medicine, University of Wisconsin-Madison for virus isolation by inoculating with Vero 76 cells. Approval to obtain the de-identified clinical sample was reviewed by the Human Subjects Institutional Review Boards at the University of Wisconsin-Madison.

Viruses. SARS-CoV-2 isolates were propagated in VeroE6 cells in Opti-MEM I (Invitrogen) containing 0.3% bovine serum albumin (BSA) and 1 µg of L-1-tosylamide-2-phenylethyl chloromethyl ketone (TPCK) treated-trypsin/ml or in Vero 76 cells in MEM supplemented with 2% FCS at 37 °C.

All experiments with SARS-CoV-2 were performed in enhanced biosafety level 3 (BSL3) containment laboratories at the University of Tokyo, which are approved for such use by the Ministry of Agriculture, Forestry, and Fisheries, Japan, or in enhanced BSL3 containment laboratories at the University of Wisconsin-Madison, which are approved for such use by the Centers for Disease Control and Prevention and by the US Department of Agriculture.

Animal experiments. The sample sizes for the hamster studies were chosen because they have previously been shown to be sufficient to evaluate a significant difference among groups (2-4). No method of randomization was used to determine how the animals were allocated to the experimental groups and processed in this study. The investigator was not blinded to the group allocation during the experiments or when assessing the outcome.

All experiments with hamsters were performed in accordance with the Science Council of Japan's Guidelines for Proper Conduct of Animal Experiments and the guidelines set by the Institutional Animal Care and Use Committee at the University of Wisconsin-Madison. The protocol was approved by the Animal Experiment Committee of the Institute of Medical Science, the University of Tokyo (approval number PA19-75) and the Animal Care and Use Committee of the University of Wisconsin-Madison (protocol number V00806).

Growth kinetics of virus in cell culture. Cultures of VeroE6, VeroE6/TMPRSS2, Calu-3, A549, NCI-H322, and NCI-H358 cells grown on 12-well plates were infected in triplicate with virus at a multiplicity of infection (MOI) of 0.05. The inoculum was removed after 60 min of incubation at 37 °C, and the cells were further incubated at 37 °C. Samples were collected at 2, 24, 48, and 72 h post-infection. Virus titers in the cell supernatant were determined by use of a plaque assay in VeroE6/TMPRSS2 cells.

Electron microscopy. VeroE6/TMPRSS2 cells were infected with virus at an MOI of 1. At 22 h post-infection, the infected cells were fixed with 2.5% glutaraldehyde in 0.1 M cacodylate buffer (pH 7.4) on ice for 1 h. After being washed with the same buffer, the cells were postfixed with 2% osmium tetraoxide on ice for 1 h. The samples were then dehydrated with a series of ethanol gradients followed by propylene oxide, and embedded in epoxy resin (Epon 812; TAAB). Thin sections (50-nm-thick) were stained with uranyl acetate and lead citrate, and examined in a Tecnai F20 electron microscope (Thermo Fisher Scientific) at 200 kV.

STEM tomography and image analysis. Semithin sections (250-nm-thick) were prepared from the same plastic block as was used for the ultrathin sections. The semithin sections were stained with uranyl acetate and lead citrate and then coated with carbon on both sides with a VE-2030 vacuum (Vacuum Device, Ibaraki, Japan). Before imaging, plasma cleaning was performed with the plasma cleaner model 1020 (Fischione Instruments, Export, PA). The tilt series was recorded with a 200-kV field emission STEM (Tecnai F20, Thermo Fisher Scientific) by using an annular dark-field detector (Fischione, Export, PA). The digital images were taken with a 2cosθ° increment over a ± 50 range with a pixel size ranging from 0.25 to 1 nm. Tomograms were reconstructed by using the simultaneous iterative reconstruction technique and Inspect3D software (Thermo Fisher Scientific). The volume segmentation, visualization, snap-shot capture, movie creation, and slab slice creation were performed with the Avizo 6.2 image processing package (Visualization Science Group, Burlington, MA, USA). Segmentation was performed based on the density and morphological characteristics of the envelope or other structures by using both automatic (the 'magic wand') and manual (the 'brush') tools.

Experimental infection of Syrian hamsters. One-month-old female Syrian hamsters (Japan SLC Inc., Shizuoka, Japan) and 7- to 8-month-old female Syrian hamsters (Envigo, Indianapolis, IN, USA) were used in this study. Baseline body weights were measured before infection. Under ketamine-xylazine anesthesia, four hamsters per group were inoculated with $10^{5.6}$ PFU (in 110 µl) or with 10^3 PFU (in 110 µl) of SARS-CoV-2/UT-NCGM02/Human/2020/Tokyo via a combination of the intranasal (100 µl) and ocular (10 µl) routes. Body weight was monitored daily for 14 days.

For virological and pathological examinations, two, four, or five hamsters per group were infected with $10^{5.6}$ PFU (in 110 µl) or with 10^3 PFU (in 110 µl) of the virus via a combination of the intranasal and ocular routes; 3, 6, and 10 days post-infection, the animals were euthanized and their organs (nasal turbinates, trachea, lungs, eyelids, brain, heart, liver, spleen, kidneys, jejunum, colon, and blood) were collected.

For the re-infection experiments, three hamsters per group were infected with $10^{5.6}$ PFU (in 110 µl) or with 10^3 PFU (in 110 µl) of UT-NCGM02 or PBS (mock) via a combination of the intranasal and ocular routes. On day 20 post-infection, these animals were re-infected with $10^{5.6}$ PFU of the virus via a combination of the intranasal and ocular routes. On day 4 after re-infection, the animals were euthanized, and the virus titers in the nasal turbinates, trachea, and lungs were determined by means of plaque assays in VeroE6/TMPRSS2 cells.

For the passive transfer experiments, eight hamsters were infected with $10^{5.6}$ PFU (in 110 µl) or with 10^3 PFU (in 110 µl) of UT-NCGM02 via a combination of the intranasal and ocular routes. Serum samples were collected from these infected hamsters on day 38 or 39 post-infection, and were pooled. Control serum was obtained from uninfected age-matched hamsters. Three hamsters per group were inoculated intranasally with 10^3 PFU of UT-NCGM02. On day 1 or 2 post-infection, hamsters were injected intraperitoneally with the post-infection serum or control serum (2 ml per hamster). The animals were euthanized on day 4 post-infection, and the virus titers in the nasal turbinates and lungs were determined by means of plaque assays in VeroE6/TMPRSS2 cells.

Micro-CT imaging. One-month-old male and female Syrian hamsters (Japan SLC Inc., Shizuoka, Japan) were used in this study. Three hamsters per group were inoculated with $10^{5.6}$ PFU or with 10^3 PFU of SARS-CoV-2/UT-NCGM02/Human/2020/Tokyo via a combination of the intranasal and ocular routes. Respiratory organs of the animals were imaged using an *in vivo* micro-CT scanner (CosmoScan FX; Rigaku Corporation, Japan) until 20 days post-infection. Under ketamine-xylazine anesthesia, the animals were placed in the image chamber and were scanned for 2 min at 90 kV,

88 μA, FOV 45 mm, and pixel size 90.0 μm. After scanning, the lung images were reconstructed by using the CosmoScan Database software of the micro-CT (Rigaku Corporation, Japan) and analyzed by using the manufacturer-supplied software.

Qualitative and semi-quantitative visual image analysis was performed by using a CT severity score adapted from a human scoring system (5). No method of randomization was used for the CT image analysis, and the scorer was not blinded to the group allocation or the time of the CT scan following inoculation. Visual observations of lung abnormalities in each of the 5 lung lobes were scored from 0–4 depending on severity. Each lung lobe was analyzed for degree of involvement and scored as 0 (none, 0%), 1 (minimal, 1%–25%), 2 (mild, 26%–50%), 3 (moderate, 51%–75%), or 4 (severe, 76%–100%). Scores for the 5 lung lobes were summed to obtain a total severity score of 0–20, reflecting the severity of abnormalities in infected hamsters compared to control hamsters.

Pathological examination. Excised tissues of animal organs were fixed in 4% paraformaldehyde phosphate buffer solution, and processed for paraffin embedding. The paraffin blocks were cut into 3-μm-thick sections and then mounted on silane-coated glass slides. One section from each tissue sample was stained using a standard hematoxylin and eosin procedure; another was processed for immunohistochemical staining with a rabbit polyclonal antibody for SARS-CoV-1 nucleoprotein (Prospec; ANT-180), which cross-reacts with SARS-CoV-2 nucleocprotein. Specific antigen-antibody reactions were visualized by means of 3,3'-diaminobenzidine tetrahydrochloride staining using the Dako Envision system (Dako Cytomation).

Virus neutralization assay. Thirty-five microliters of virus (140 tissue culture infectious dose 50) was incubated with 35 μl of two-fold serial dilutions of sera for 1 h at room temperature, and 50 μl of the mixture was added to confluent VeroE6/TMPRSS2 cells in 96-well plates, and incubated for 1 h at 37 °C. After the addition of 50 μl of DMEM containing 5% FCS, the cells were further incubated for 3 days at 37 °C. Viral cytopathic effects (CPE) were observed under an inverted microscope and virus neutralization titers were determined as the reciprocal of the highest serum dilution that completely prevented the CPE.

Enzyme-linked immunosorbent assay (ELISA). The ELISA was performed using a recombinant receptor-binding domain (RBD) protein with a C-terminal hexahistidine-tag purified (6) by using TALON metal affinity resin from Expi293F cells (Thermo Fisher Scientific). The ELISA plates were coated overnight at 4 ºC with 50 μl of the RBD protein at a concentration of 2 μg/ml in phosphate-buffered saline (PBS). After blocking the plates with PBS containing 0.1% Tween 20 (PBS-T) and 3% milk powder, the plates with incubated in duplicate with heat-inactivated (56 ºC for 30 minutes) serum diluted in PBS-T with 1% milk powder. After a 4-hour incubation at room temperature, the plates were washed with PBS-T three times and then incubated with a hamster IgG secondary antibody conjugated with horseradish peroxidase (Invitrogen; 1:7,000 dilution in PBS-T with 1% milk powder). After a 1-hour incubation with the secondary antibody, the plates were washed three times with PBS-T and then developed with SigmaFast o-phenylenediamine dihydrochloride solution (Sigma). After a 10-minute incubation, the reaction was stopped with the addition of 3 M hydrochloric acid. The absorbance was measured at a wavelength of 490 nm (OD_{490}). Background measurements from day 0 plasma were subtracted from the day 24 plasma for each dilution. IgG antibody titer was defined as the highest plasma dilution with an OD_{490} cut-off value of 0.15.

Statistical analysis. Data are expressed as the mean ± SD. For hamster organ titers, statistical analyses were performed using two-tailed unpaired Student's t-tests; *p*-values of < 0.05 were considered to be statistically significant. For the analysis of the body weight data, we also performed a linear mixed effects analysis. As fixed effects, we used the infection groups, and the time of measurement (with an interaction term between those fixed effects). As random effects, we had intercepts for the individual animals. We used the R statistical package (www.r-project.org), lme4 (7), and the EMMeans package (https://CRAN.R-project.org/package=emmeans). For the group comparisons, we compared the low- and high-dose values to the mock group. The *p*-values were adjusted using Holm's method, and in all cases, we considered the differences significant if the *p*-values were <0.05.

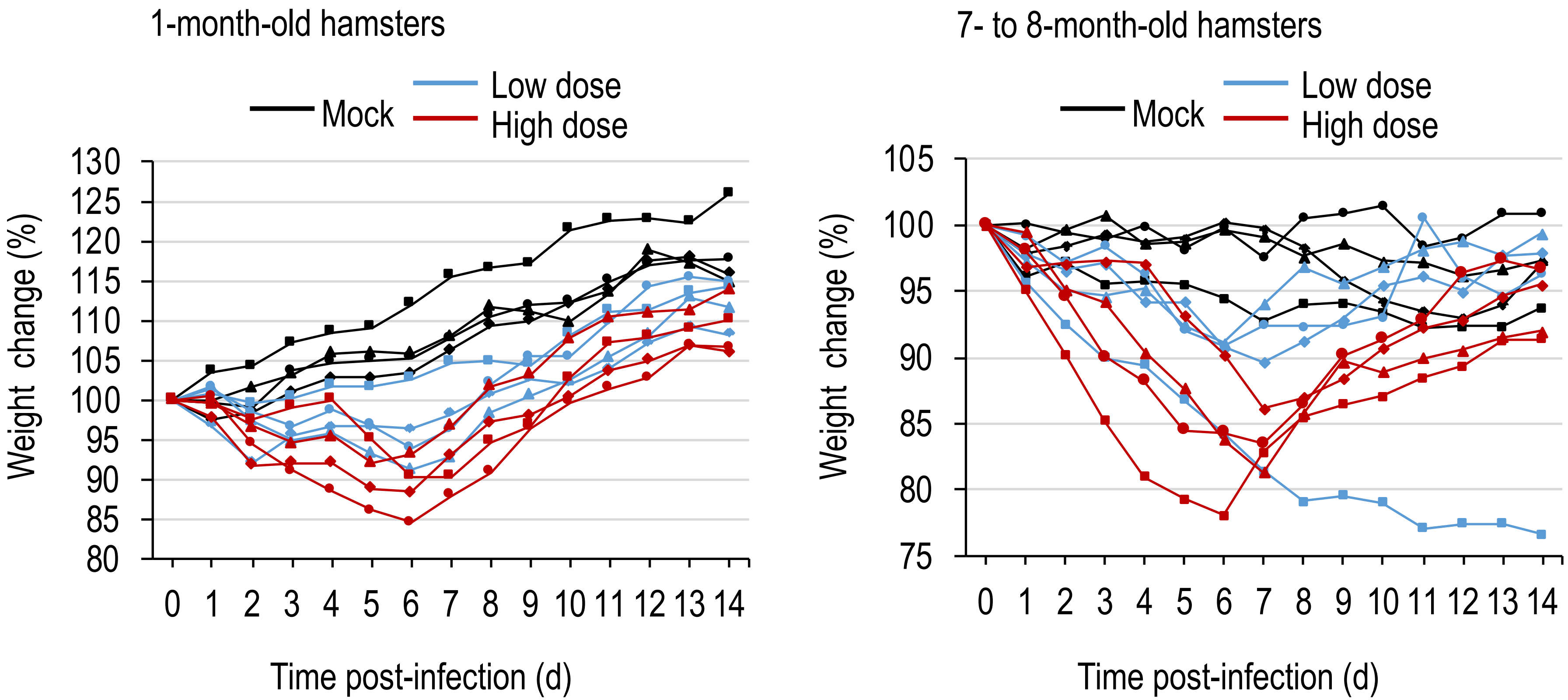

Figure S1. Body weight changes in infected Syrian hamsters. Syrian hamsters were inoculated with $10^{5.6}$ PFU (in 110 µl) or with 10^{3} PFU (in 110 µl) of SARS-CoV-2/UT-NCGM02/Human/2020/Tokyo or with PBS (mock) via a combination of the intranasal (100 µl) and ocular (10 µl) routes. Body weights of virus-infected (*n*=4) and mock-infected hamsters (*n*=4) were monitored daily for 14 days.

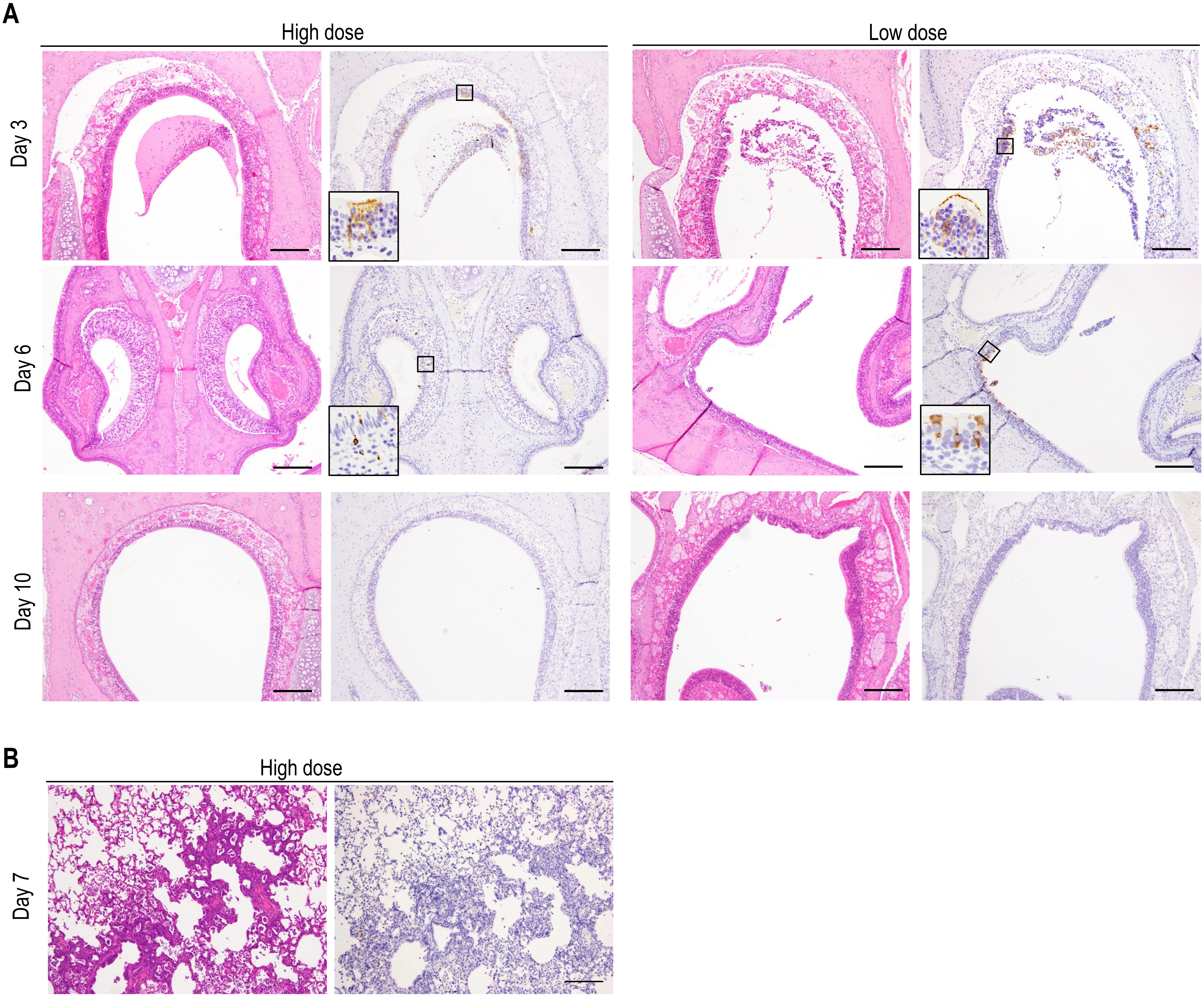

Figure S2. Pathological findings in infected Syrian hamsters. (A) Histopathological examination of the nasal turbinates of infected hamsters. Syrian hamsters were inoculated with $10^{5.6}$ PFU (in 110 µl) or with 10^3 PFU (in 110 µl) of SARS-CoV-2/UT-NCGM02/Human/2020/Tokyo via a combination of the intranasal (100 µl) and ocular (10 µl) routes. Syrian hamsters infected with the high or low dose were euthanized on days 3, 6, and 10 post-infection for pathological examinations (*n*=2, except for 1 in the high-dose group on day 10). Shown are representative pathological findings in the nasal turbinates of virus-infected hamsters on days 3, 6, and 10 post-infection [left panels, hematoxylin and eosin (HE) staining; right panels, immunohistochemistry for SARS-CoV-2 viral antigen detection]. Scale bars, 200 µm. (B) Pathological findings in the lungs of a Syrian hamster that exhibited severe weight loss. Of the animals inoculated with $10^{5.6}$ PFU of the virus, one was humanely euthanized on day 7 post-infection because it had lost > 25% of its initial body weight at this timepoint. Shown are representative pathological findings in the lungs [left panels, hematoxylin and eosin (HE) staining; right panels, immunohistochemistry for SARS-CoV-2 viral antigen detection]. Scale bars, 200 µm.

Table S1. Virus replication in 1-month-old hamsters infected with SARS-CoV-2*

	Virus titers (log_{10} PFU/g) of animals infected with SARS-CoV-2																							
	Day 3								Day 6								Day 10							
	High dose ($10^{5.6}$ PFU)				Low dose (10^{3} PFU)				High dose ($10^{5.6}$ PFU)				Low dose (10^{3} PFU)				High dose ($10^{5.6}$ PFU)				Low dose (10^{3} PFU)			
Animal ID	#1	#2	#3	#4	#5	#6	#7	#8	#9	#10	#11	#12	#13	#14	#15	#16	#17	#18	#10	#20	#21	#22	#23	#24
Nasal turbinate	8.4	8.8	8	8.4	8.5	7.8	8.2	8.4	6.2	5.1	4.5	6.7	5.3	4.3	4.3	5	-†	-	-	-	1.9	-	-	-
Trachea	5.4	5.9	5.9	6.1	4.5	5.9	4.7	6.1	3.2	2.5	2.5	4.7	3.8	-	-	2.3	-	-	-	2.4	-	-	-	-
Lung right cranial and accessory lobes	8.2	8.4	8	8.4	8.6	8.1	9	9.3	5.2	4.4	4.4	5.7	5.5	5	4.4	3.9	-	-	-	-	-	-	-	-
Lung right middle lobe	8	8.3	8.3	8.6	8.2	8.3	8.4	8.8	6	4.6	5.1	6.4	6.3	4.9	5.6	5.7	-	-	-	-	-	-	-	-
Lung right caudal lobe	8	8.7	8.5	8.2	8.5	8.6	8.4	8.5	5.3	5.5	5.3	6	5.2	4.9	4.4	5.2	-	-	-	-	-	-	-	-
Lung left lobe	8	8.6	8.2	8.6	8.4	8.6	8.2	8.5	5.2	5.1	5	6.2	6.2	5.2	4.7	5.4	-	-	-	-	-	-	-	-
Eyelid	1.7	-	2.2	-	-	-	-	-	-	-	-	-	-	-	-	-	-	-	-	-	-	-	-	-
Brain	2	6.5	3.8	1.9	2.5	2.7	1.9	1.5	-	-	-	-	-	-	-	-	-	-	-	-	-	-	-	-
Heart	-	1.8	-	-	-	-	-	-	-	-	-	-	-	-	-	-	-	-	-	-	-	-	-	-
Liver	1.9	-	1.8	1.7	-	1.6	1.5	-	-	-	-	-	-	-	-	-	-	-	-	-	-	-	-	-
Spleen	-	-	-	1.9	-	1.9	-	-	-	-	-	-	-	-	-	-	-	-	-	-	-	-	-	-
Kidney									-	-	-	-	-	-	-	-	-	-	-	-	-	-	-	-
Jejunum	-	-	-	-	-	-	-	-	-	-	-	-	-	-	-	-	-	-	-	-	-	-	-	-
Colon	-	-	-	-	-	-	-	-	-	-	-	-	-	-	-	-	-	-	-	-	-	-	-	-
Blood	-	-	-	-	-	-	-	-	-	-	-	-	-	-	-	-	-	-	-	-	-	-	-	-

*Syrian hamsters were inoculated with $10^{5.6}$ PFU (in 110 µl) or with 10^{3} PFU (in 110 µl) of SARS-CoV-2/UT-NCGM02/Human/2020/Tokyo via a combination of the intranasal (100 µl) and ocular (10 µl) routes. Four Syrian hamsters per group were euthanized on days 3, 6, and 10 post-infection for virus titration.

†-, virus not detected.

Table S2. Virus replication in 7 to 8-month-old hamsters infected with SARS-CoV-2[*]

	Virus titers (log_{10} PFU/g) of animals infected with SARS-CoV-2																							
	Day 3								Day 6								Day 10							
	High dose				Low dose				High dose				Low dose				High dose				Low dose			
	($10^{5.6}$ PFU)				(10^3 PFU)				($10^{5.6}$ PFU)				(10^3 PFU)				($10^{5.6}$ PFU)				(10^3 PFU)			
Animal ID	#1	#2	#3	#4	#5	#6	#7	#8	#9	#10	#11	#12	#13	#14	#15	#16	#17	#18	#10	#20	#21	#22	#23	#24
Nasal turbinate	6.9	6.9	6.8	5.3	8.3	6.3	7.7	7.5	3.7	2.3	2.4	2.3	2.4	2.4	2.5	2.8	-†	-	-	-	-	-	-	-
Trachea	4.6	3.7	6.1	6.1	6	6	6.5	6.6	2.8	3.2	2.9	3.1	2.8	2.2	2.7	3.2	-	-	-	-	-	-	-	-
Lung right cranial and accessory lobes	7.5	8	7.7	7.4	6.6	4.2	7.5	8	5.4	4.3	4.4	3.2	2.9	2.6	2.9	2.2	-	-	-	-	-	-	-	-
Lung right middle lobe	7.3	7.9	7.6	7.6	7.3	4.1	7.7	8	5	4.8	4.7	3.4	3.0	3.0	3.6	2.5	-	-	-	-	-	-	-	-
Lung right caudal lobe	7.6	7.3	7.3	7.4	6.6	3.6	7.6	8.4	4.6	3.5	4.7	4.0	3.2	3.2	3.4	2.3	-	-	-	-	-	-	-	-
Lung left lobe	7.3	8.6	7.6	6.7	8.1	8.4	7.4	8.1	4.7	4.5	4.9	3.9	4.3	1.9	2.4	2.2	-	-	-	-	-	-	-	-
Eyelid	-	-	-	-	-	-	-	-	-	-	-	-	-	-	-	-	-	-	-	-	-	-	-	-
Brain	-	-	-	2.3	-	-	-	-	-	-	-	-	-	-	-	-	-	-	-	-	-	-	-	-
Heart	-	-	-	-	-	-	-	-	-	-	-	-	-	-	-	-	-	-	-	-	-	-	-	-
Liver	-	-	-	-	-	-	-	-	-	-	-	-	-	-	-	-	-	-	-	-	-	-	-	-
Spleen	-	-	-	-	-	-	-	-	-	-	-	-	-	-	-	-	-	-	-	-	-	-	-	-
Kidney	-	-	-	-	-	-	-	-	-	-	-	-	-	-	-	-	-	-	-	-	-	-	-	-
Jejunum	-	-	-	-	-	-	-	-	-	-	-	-	-	-	-	-	-	-	-	-	-	-	-	-
Colon	-	-	-	-	-	-	-	-	-	-	-	-	-	-	-	-	-	-	-	-	-	-	-	-
Blood	-	-	-	-	-	-	-	-	-	-	-	-	-	-	-	-	-	-	-	-	-	-	-	-

[*]Syrian hamsters were inoculated with $10^{5.6}$ PFU (in 110 µl) or with 10^3 PFU (in 110 µl) of SARS-CoV-2/UT-NCGM02/Human/2020/Tokyo via a combination of the intranasal (100 µl) and ocular (10 µl) routes. Four Syrian hamsters per group were euthanized on days 3, 6, and 10 post-infection for virus titration.

†-, virus not detected.

Table S3. Effect of convalescent serum on the replication of SARS-CoV-2 in hamsters*

Serum was administered to recipient hamsters on:	Passively transferred serum	Neutralizing antibody titer in serum†	Animal ID	Neutralizing antibody titer in serum collected from recipient hamsters on day 4 post-infection‡	Virus titers (log_{10} PFU/g) in:	
					Nasal turbinate	Lung
Day 1 post-infection	Infected hamster serum	640	#1	40	6.0	5.7
			#2	80	5.8	5.2
			#3	80	6.6	5.0
	Uninfected hamster serum	<10	#4	<10	7.5	8.2
			#5	<10	7.6	8.2
			#6	<10	7.0	8.2
Day 2 post-infection	Infected hamster serum	640	#7	160	5.7	4.6
			#8	40	6.0	5.0
			#9	<10	7.1	8.0
	Uninfected hamster serum	<10	#10	<10	7.0	7.8
			#11	<10	6.6	7.7
			#12	<10	6.6	7.8

*Syrian hamsters (n=3 for each group) were inoculated intranasally with 10^3 PFU of SARS-CoV-2/UT-NCGM02/Human/2020/Tokyo. On day one or two post-infection, the hamsters were injected intraperitoneally with post-infection serum or normal uninfected serum. Animals were euthanized on day 4 post-infection for virus titration.

†Viral neutralization titers against inoculated virus; Sera were collected from eight hamsters on day 38 or 39 post-infection and then pooled.

‡Viral neutralization titers against inoculated virus; Sera were collected on day 4 post-infection.

Movie S1. Three-dimensional models of the RNP (red) within a virion.

Movie S2. Three-dimensional models of the RNP (purple) within a virion.

Movie S3. Three-dimensional images were obtained by integrating CT images of the lungs of an uninfected hamster. The trachea and bronchi are colored in blue.

Movie S4. Three-dimensional images were obtained by integrating CT images of the lungs of an infected hamster at day 2 post-infection. The trachea and bronchi are colored in blue.

Movie S5. Three-dimensional images were obtained by integrating CT images of the lungs of an infected hamster at day 7 post-infection. The pneumothorax is colored in red. The trachea and bronchi are colored in blue.

Movie S6. Three-dimensional images were obtained by integrating CT images of the lungs of an infected hamster at day 10 post-infection. The trachea and bronchi are colored in blue.

Movie S7. Three-dimensional images were obtained by integrating CT images of the lungs of an infected hamster at day 16 post-infection. The trachea and bronchi are colored in blue.

SI References

1. Matsuyama S*, et al.* (2020) Enhanced isolation of SARS-CoV-2 by TMPRSS2-expressing cells. *Proc Natl Acad Sci U S A* 117(13):7001-7003.
2. Lamirande EW*, et al.* (2008) A live attenuated severe acute respiratory syndrome coronavirus is immunogenic and efficacious in golden Syrian hamsters. *J Virol* 82(15):7721-7724.
3. Roberts A*, et al.* (2006) Therapy with a severe acute respiratory syndrome-associated coronavirus-neutralizing human monoclonal antibody reduces disease severity and viral burden in golden Syrian hamsters. *J Infect Dis* 193(5):685-692.
4. Roberts A*, et al.* (2005) Severe acute respiratory syndrome coronavirus infection of golden Syrian hamsters. *J Virol* 79(1):503-511.
5. Chung M*, et al.* (2020) CT Imaging Features of 2019 Novel Coronavirus (2019-nCoV). *Radiology* 295(1):202-207.
6. Amanat F*, et al.* (2020) A serological assay to detect SARS-CoV-2 seroconversion in humans. *Nat Med*.
7. Bates D, Machler M, Bolker BM, & Walker SC (2015) Fitting Linear Mixed-Effects Models Using lme4. *J Stat Softw* 67(1):1-48.
